# Multiple functions of autophagy in vascular calcification

**DOI:** 10.1186/s13578-021-00639-9

**Published:** 2021-08-16

**Authors:** Xin Zhou, Sui-Ning Xu, Shu-Tong Yuan, Xinjuan Lei, Xiaoying Sun, Lu Xing, Hui-Jin Li, Chun-Xia He, Wei Qin, Dong Zhao, Peng-Quan Li, Edward Moharomd, Xuehong Xu, Hui-Ling Cao

**Affiliations:** 1 Shaanxi Key Laboratory of Ischemic Cardiovascular Disease, Shaanxi Key Laboratory of Brain Disorders, Institute of Basic & Translational Medicine, Xi’an Medical University, Xi’an, 710021 Shaanxi China; 2 Department of Cardiology, The First Affiliated Hospital, Xi’an Medical University, Xi’an, 710021 Shaanxi China; 3 Laboratory of Cell Biology, Genetics and Developmental Biology, Shaanxi Normal University College of Life Sciences University Hospital Medical Center, Xi’an, 710062 Shaanxi China; 4 College of Pharmacy, Shaanxi University of Chinese Medicine, Xianyang, 712046 Shaanxi China; 5 Johns Hopkins University School of Medicine, Baltimore, MD 21205 USA

**Keywords:** Vascular calcification, Autophagy/mitophagy, Osteoblastic differentiation of VSMCs, Osteogenesis, AMPK/mTOR, HIF-1a/PDK4, EphrinB2, GAS6/AXL

## Abstract

**Background:**

Vascular calcification is a closely linked to cardiovascular diseases, such as atherosclerosis, chronic kidney disease, diabetes, hypertension and aging. The extent of vascular calcification is closely correlate with adverse clinical events and cardiovascular all-cause mortality. The role of autophagy in vascular calcification is complex with many mechanistic unknowns.

**Methods:**

In this review, we analyze the current known mechanisms of autophagy in vascular calcification and discuss the theoretical advantages of targeting autophagy as an intervention against vascular calcification.

**Results:**

Here we summarize the functional link between vascular calcification and autophagy in both animal models of and human cardiovascular disease. Firstly, autophagy can reduce calcification by inhibiting the osteogenic differentiation of VSMCs related to ANCR, ERα, β-catenin, HIF-1a/PDK4, p62, miR-30b, BECN1, mTOR, SOX9, GHSR/ERK, and AMPK signaling. Conversely, autophagy can induce osteoblast differentiation and calcification as mediated by CREB, degradation of elastin, and lncRNA H19 and DUSP5 mediated ERK signaling. Secondly, autophagy also links apoptosis and vascular calcification through AMPK/mTOR/ULK1, Wnt/β-catenin and GAS6/AXL synthesis, as apoptotic cells become the nidus for calcium-phosphate crystal deposition. The failure of mitophagy can activate Drp1, BNIP3, and NR4A1/DNA‑PKcs/p53 mediated intrinsic apoptotic pathways, which have been closely linked to the formation of vascular calcification. Additionally, autophagy also plays a role in osteogenesis by regulating vascular calcification, which in turn regulates expression of proteins related to bone development, such as osteocalcin, osteonectin, etc. and regulated by mTOR, EphrinB2 and RhoA. Furthermore, autophagy also promotes vitamin K2-induced MC3T3 E1 osteoblast differentiation and FGFR4/FGF18- and JNK/complex VPS34–beclin-1-related bone mineralization via vascular calcification.

**Conclusion:**

The interaction between autophagy and vascular calcification are complicated, with their interaction affected by the disease process, anatomical location, and the surrounding microenvironment. Autophagy activation in existent cellular damage is considered protective, while defective autophagy in normal cells result in apoptotic activation. Identifying and maintaining cells at the delicate line between these two states may hold the key to reducing vascular calcification, in which autophagy associated clinical strategy could be developed.

## Introduction

Regarded as a common pathological manifestation of patients with atherosclerosis, chronic kidney disease (CKD), diabetes, hypertension, postmenopausal syndrome, aortic stenosis [1–3] and the aging population [4], vascular calcification (VC) significantly correlated with cardiovascular and all-cause mortality, via deleterious mechanical effects on vascular compliance and vasomotion [5, 6]. Pathological abnormalities of VC may cause further adverse cardiovascular events and even induce death. The importance of VC to human health has attracted more attention, but the molecular mechanism of VC is under further investigation.

As a fundamental process for degradation and recycling of exhausted cellular components prevalent in eukaryotes, autophagy has recently been recognized in various physiological and pathological events, which presents a unique mechanism of self-regulating/cleaning [7–9]. Briefly, as a survival mechanism within an intracellular degradation system, autophagy process is composed of numerous chronological steps including sequestration, transport to lysosomes, degradation of cytoplasmic components, and utilization of degradation products. This self-degradative process tightly associates with both physiological and pathological status within normal embryonic and postnatal development in the behaviors of microautophagy, macroautophagy, chaperone-mediated autophagy and other new discovered manners [10].

The autophagic mechanism tightly associates with critical signaling pathways including PI3K/AKT, MAPK/Erk1/2, mTOR, AMPK, p53, HIF-1α/PDK4, β-catenin, ULK and Atg involved in regulation of the cellular autophagy [11, 12]. It has been intensely understood that autophagy functions in the cardiovascular diseases [13, 14], and the autophagic phenotype associated to vascular smooth muscle linking to steogenic differentiation [15], apoptosis [16], inflammation [17], Fibroblast Growth Factor 23 (FGF23)-Klotho [17, 18], Matrix Vesicle (MV) release [19], and oxidative stress [20] physiological or pathological conditions.

In both tumorigenesis and cardiovascular pathology, calcium (Ca^2+^) is considerate to be an essential constituent vital to the healthy physiology and disease pathology of both tumor cells and myocytes [21–24]. In regulation of cellular proliferation and apoptotic death, AKT/AMPK pathway control the cell cycle by targeting on critical point of G2/S transition functioning along with Ras and Cyclin D1. With involvement of autophagic proteins including Beclin, LC3-1 and LC3-II, up-regulations of AMPK, phospholated pAKT and pmTOR powerfully link Akt/mTOR associated autophagy to osteogenic differentiation of human mesenchymal stem cells [25].

Recently, it confirmed that the VC process accompanies on expression alternation of vascular smooth muscle cells (VSMCs) contractile phenotype-related factors such as α-SMA, calponin-1, SM22α and others along with the imbalance of a variety of calcification promoting factors including ALP, Runx2, BMPs, OCN, Collagen I and their inhibitors [26, 27]. Osteoblasts derived from VSMCs and mesenchymal stromal cells (MSCs) are regulated by autophagy [28, 29] and promotes transition of calcification signals for mineralization in the vessel wall of the vascular structure [30–32]. Autophagy within the physiological range functions protective effect but pathologic autophagy generates excessively or less activation. In this paper, we elucidate that the clarification on the mechanism of autophagy regulated VC would provide valuable information for developing diagnostic strategy and anti-VC drug design targeting on autophagy.

## Autophagy affects vascular calcification by interfering with the osteogenic differentiation of VSMCs

As defined as an active, highly controllable mineral deposition process, the pathological changes of VC involve intima and middle layer of blood vessels, mainly VSMCs included in vessels-wall structure [33]. In the VC process, VSMCs transform from contractile phenotype to osteogenic/chondral phenotype directly or through synthetic inter-type [15]. Currently known related signaling pathways include ERα, β-catenin, HIF-1a/PDK4, p62, miR-30b, BECN1, mTOR, SOX9, GHSR/ERK, AMPK, Elastin [34–39]. The definite mechanism of osteoblastic differentiation of VSMCs is critical for vascular calcification.

### Autophagy reduces calcification by inhibiting the osteogenic differentiation of VSMCs

Liang, et al. proved that long non‑coding RNA‑ANCR promoted the expression of LC3 and Atg5 in β‑GP‑induced VSMCs, and inhibited osteoblastic differentiation of VSMCs. The ANCR may attenuate arterial calcification through activating autophagy that inhibits osteogenic differentiation of VSMCs [40]. The autophagy inhibitor 3-MA or knockout of Atg5 increased calcium deposition, whereas the autophagy inducer valproic acid reduced VSMC calcification [31]. Yuan and colleagues approved that oestrogen inhibited the osteoblastic differentiation of VSMCs by promoting autophagy through the ERα but not ERβ signaling pathway [34].

**Fig. 1:**
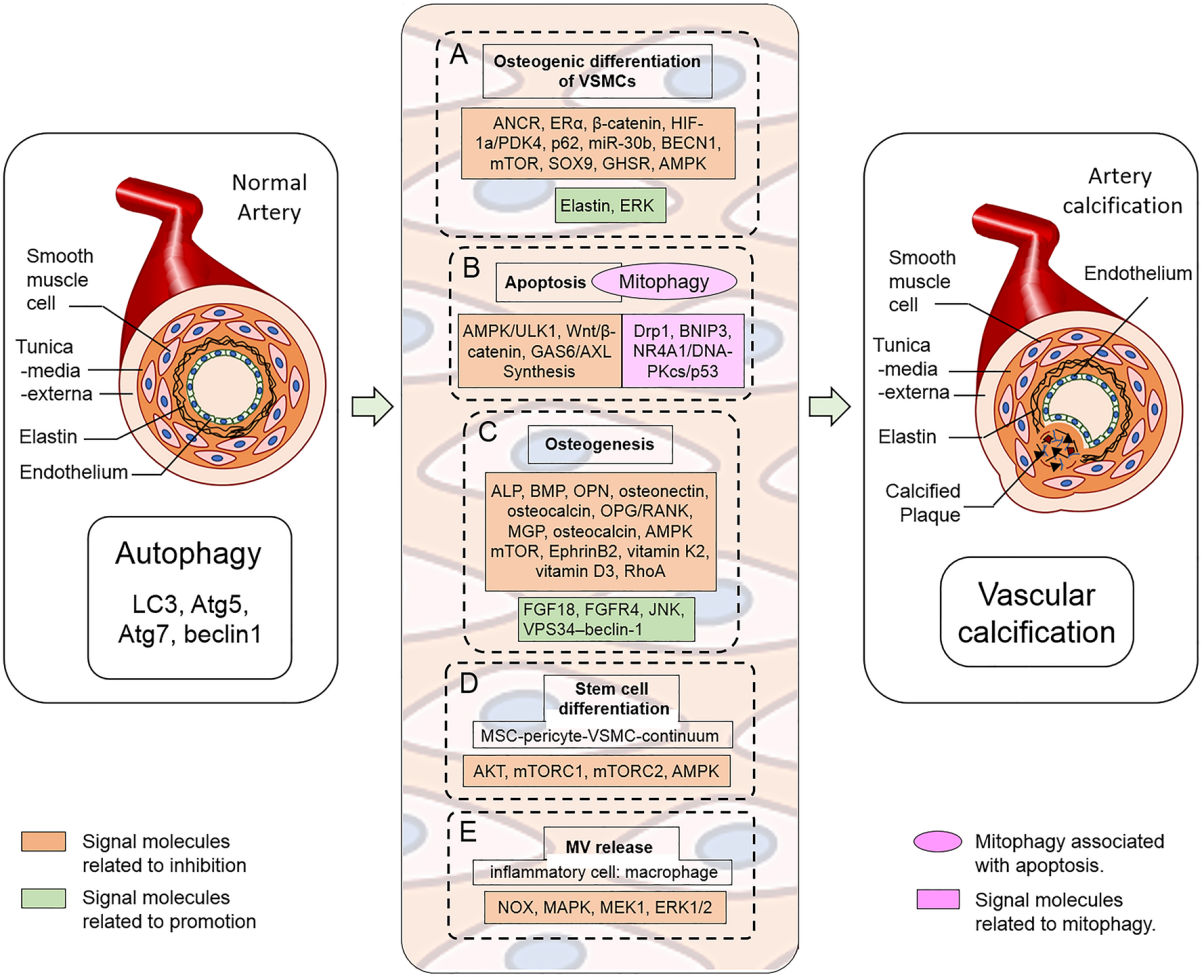
Signaling molecules linking autophagy to vascular calcification. **A** Autophagy affects VC by interfering with the osteogenic differentiation of VSMCs. **B** Autophagy may affect VC by inhibiting apoptosis. **C** VC is a tightly cell-regulated pathological process that resembles osteogenesis. **D** The effect of autophagy regulation in stem cells on VC. **E** MV involved in autophagy regulation of VC

Statins display various protective effects against VSMC proliferation and inflammation in cardiovascular remodeling [41] and inhibit calcification of atherosclerotic plaques in the apoE-deficient mice [42]. Results from clinical trials suggest an association of statins usage with slow progression of calcific aortic stenosis, and coronary artery calcification [43, 44]. Liu and colleagues found inhibitory effect of atorvastatin on calcification is caused by inducing autophagy by using 3-MA, chloroquine, NH_4_Cl and bafilomycin A1. Their data approved that atorvastatin can protect VSMC differentiation from TGF-β1-stimulated calcification through suppression of β-catenin pathway [35]. Their data are consistent with the recently results that atorvastatin could reduce arterial calcification and plasma calcium concentration [45] (Fig. 1).

Recently, it is approved that AGEs could increase alkaline phosphatase (ALP) and accelerate the calcification of VSMCs [46]. AGE-BSA treatment on VSMCs improved the expression of PDK4 via HIF-1α upregulation. The AGE-BSA incubation promoted expression increase of LC3-II and decrease of p62 protein levels. The treatment could enhance autophagic flux mediated by mRFP-GFP-LC3 adenovirus, make co-localization of LC3-II and LAMP-1, and eventually augmenter the number of autophagosome under TEM. HIF-1a/PDK4 pathway was activated in the process of AGEs-induced autophagy of VSMCs, which reduced the expression of the Runt-related transcription factor (RUNX2) and presented protective effects against VC induced by AGEs [36].

Some miRNAs are implicated in proliferation, development, and function of VSMCs, and directly involved in pathological calcification [47]. miR-30b regulates the Runx2 expression and plays an important role in VC as a common feature in patients with CKD [48]. More studies have highlighted the central role of miR-30b in high Pi level-induced autophagy via the regulation of BECN1, which suggested miR-30b as possible target for the treatment of vascular diseases [49]. Li, et al.clarified that restoring miR-30b can promote autophagy while inhibiting VC by modulating mTOR signaling pathway in β‐glycerophosphate induced VSMCs. miR‐30b negative regulates SOX9 while restoring miR‐30b in cell can increase the mitochondrial membrane potential (MMP) in β‐glycerophosphate‐induced VSMCs [37].

Hormonal ghrelin prevents osteoblastic transformation and mineralization of VSMCs mediated by GHSR/ERK signaling pathway [38]. Ghrelin application increases the expression of LC3 and beclin1 indicated autophagy, while 3-MA delays the ameliorative effect of ghrelin on VC. The protein levels of p-AMPK are promoted by the hormonal treatment, and AMPK inhibitor, compound C blocks the effect of ghrelin on VC and autophagy. In animal model, the hormone promotes autophagy in VC aorta and activates AMPK pathway meanwhile. Improved autophagy was detected following the activation of AMPK, which resulted in VC amelioration [50].

### Autophagy can promote the osteogenic differentiation of VSMCs within diverse circumstances

The co-relationship between calcification and autophagy indicates that autophagy is one target for inhibition of VSMC calcification. Different from the inhibitory effect of autophagy on the osteogenic trans-differentiation of VSMCs, indoxyl sulfate stimulates the autophagy pathway through downregulating the expression of SET domain encompassing lysine methyltransferase 7/9. Subsequently, it can induce osteoblast differentiation and matrix mineralization of VSMCs [51] (Table 1).

**Table 1:** Autophagy involved vessel calcification through inhibition and promotion cellular pathways

Functions	Targeted genes	Animal models and cell culture	Involved genes
VSMC osteogenic differentiation			
Autophagic inhibition	ANCR [34]	**Animal model** High calcitriol-induced mice model /male C57BL/6 **Cell culture** Mouse VSMCs of passages 3–8	Atg5, BMP-2, Cbfa1/Runx2, LC3-I/II
	ERα [28]	**Animal model** 6-week-old female C57/BL6 mice were ovariectomized under anaesthesia (by Nembutal 40 mg/kg i.m.). **Cell culture** Mouse vascular smooth muscle cells (mVSMCs) were acquired from 8-week-old female C57/BL6 OVX mice or mice with intact ovaries. **Patient tissue** Renal arterial samples from a total of 10 pairs of uremic patients scheduled to undergo kidney transplantation and from healthy donors	Atg5, ALP, Cbfa1/Runx2, ERα, LC3-I/II
	β-catenin [29]	**Cell culture** VSMCs from Male Sprague–Dawley (SD) rats aorta	Atg5, ALP, BMP-2, Beclin-1, Collagen I, Histone H2B, LC3, Osteocalcin, β-catenin
	HIF-1a, PDK4, p62/SQSTM1 [30]	**Cell culture** VSMCs from Male SD rats aorta	Cbfa1/Runx2, HIF-1a, LC3, PDK4, p62/SQSTM1
	BECN1 [43]	**Cell culture** VSMCs from the descending thoracic aorta of rats, 293 T cells	Atg4b, Atg5, Atg7, Atg12, Atg16, BECN1, LC3B, LC3I/II, ULK1, VPS34
	mTOR, SOX9 [31]	**Animal model** 5‐weeks‐old chronic kidney disease rats (5/6 nephrectomy) **Cell Culture** VSMC lines	Beclin-1, Cbfa1/Runx2, LC3, LC3-II, miR-30b, mTOR, p‐mTOR, SOX9, S6K1, p‐S6K1
	GHSR/ERK [32]	**Cell culture** Mouse VSMCs	ALP, Cbfa1/Runx2, ERK, p-ERK, GHSR, JNK, p-JNK, p-p38, p38
	AMPK [44]	**Animal model** 3-month-old male SD rats **Cell culture** Primary VSMCs from thoracic aortas of male SD rats	AKT, p-AKT, Beclin-1, LC3, mTOR, p-mTOR
Autophagic promotion	ERK [47]	**Animal model** 6-week-old ApoE^−/−^ mice (C57BL/6 J genetic background) **Cell culture** Human VSMCs (HA-VSMC, CRL-1999, ATCC, Manassas, VA, USA)	ALP, Beclin-1, DUSP5, ERK1/2, p-ERK1/2, LC3, LC3-I/II, mTOR, p-mTOR, p62/SQSTM1
Autophagic/ mitophagic apoptosis			
Autophagic inhibition	AMPK/mTOR/ULK1 [54]	**Cell culture** VSMCs from the aortas of 4-week-old SD rats	ALP, AMPK, p-AMPK, BMP-2, Cbfa1/Runx2, Caspase 3, Cleaved caspase 3, LC3-II, LC3-I, MGP, p62/SQSTM1, α-SMA, mTOR, ULK1, p-ULK1
	Wnt/β-catenin [55]	**Cell culture** Primary HASMCs (human aortic smooth muscle cells)	BMP-2, Beclin-1, Bax, Bak, Bcl-2, Cbfa1/Runx2, β-catenin, Caspase 9, Caspase 3, LC3-II, p-mTOR, OPN, OPG, p62/SQSTM1, α-SMA
	GAS6/AXL synthesis [56]	**Cell culture** Rat VSMCs	Axl, GAS6, LC3-II
Autophagic promotion	Drp1, BNIP3, NR4A1/DNA-PKcs/p53 [59]	**Animal model** 6-week-old male Wistar rats **Cell culture** Primary VSMCs from 6-week-old SD rat thoracic aortas	Bax, Bcl-2, BNIP3, BMP-2, Caspase 3, Cbfa1/Runx2, Drp1, DNA-PKcs, LC3, Mff, NR4A1, OPA1, p62/SQSTM1, p53, α-SMA, TOMM20, TOMM40, VDAC1
Osteogenesis			
Inhibition on osteoblast/osteoclast	AMPK mTOR [73]	**Cell culture** Primary human chondrocytes	ALP, Adiponectin, AMPK, p-AMPK (Thr172), Beclin-1, Caspase 3, Caspase 9, LC3-I, LC3-II, mTOR, p- mTOR, PARP
	EphrinB2, RhoA [74]	**Animal model** Dmp1Cre mice (Tg(Dmp1Cre)^1Jqfe^), EphrinB2-floxed (Efnb2^tm1And^) mice **Cell culture** Ocy454 cells and Kusa 4b10 cells	BNIP3, Eps8l1, Efnb2, Fam134b, Fbxo32, Klf1, Lama2, LC3-I, LC3-II, Pthlh, Peg3, RhoA, Trim63, Tspo2, Unc5a
Promotion on osteoblast/osteoclast	FGFR4, JNK, VPS34, beclin-1 [85]	**Animal model***Atg7*^fl/fl^, GFP–LC3 mouse, *Prx1*-Cre, *Col2a1*-Cre, *Fgf18-* and *Fgfr3-*knockout mice, *Fgfr4-*knockout mice **Cell culture** GFP–LC3 primary chondrocytes, *Fgf18*^+/−^ chondrocytes	Atg7, AKT, p-AKT, AMPKa, p-AMPKa, Beclin-1, p-BCL2, Collagen II, ERK1/2, p-ERK, FGFR3, FGFR4, GOLPH3, HA, Histone H3, JNK, p-JNK, p–c-JUN, LC3B, mTORC1, p-mTORC1, PDI, p38, p-p38, p62/SQSTM1, P70S6K, p-P70S6K, VPS34, 4EBP1, p-4EBP1
Regerentation (MSC-pericyte-VSMC-continuum)			
Autophagic inhibition	AKT, mTORC1, mTORC2 [89]	**Animal model** Male 10-week-old C57BL/6JRccHsd mice **Cell culture** Human bone marrow aspirates MSC	AKT, Bcl-2, Cleaved caspase 3, Calponin, Cbfa1/Runx2, Collagen IIA1, Collagen III, ERK1/2, ERK1/2^Thr202/Tyr204^, LC3B, MLCK, mTOR, Osteopontin, Osterix, p16INK4a, p62/SQSTM1, pp70S6K^Thr389^, pp70S6K^Thr421/Ser424^, pAKT^Ser473^, pAKT^Thr308^, p70-S6, Rictor, Raptor, SMA, SM22α
	AMPK [90]	**Animal model** 6-weeks-old male SD rats **Cell culture** Tenocytes isolated from 3-week-old SD rats achilles tendons	Cleaved caspase 3, Cleaved caspase 9, LC3A/B, p62/SQSTM1, p53, p21
Cellular derived MV (Macrophage inflammatory)			
Autophagic inhibition	NOX-1, MAPK, MEK1, ERK1/2 [96]	**Cell culture** Primary rat VSMC from the descending thoracic aorta of CKD-MBD, Cy/ + rat	AT1R, Annexin II, Annexin V, Annexin VI, BMP-2, Cbfa1/Runx2, CD9, CD63, CD81, p-ERK1/2, Fetuin-A, p-MEK1, Myocardin, NOX-1, NOX-4, Osteocalcin, SM22α, SOD-1, SOD-2

Low dietary potassium can induce elevation of intracellular calcium, activate intracellular calcium signaling-mediated CREB and autophagy, and further promote VSMC osteoblast differentiation and calcification [52]. The occurrence and development of VC symptom caused by autophagy were linked to autophagy-induced degradation of elastin [39].

Interestingly, in osteogenic differentiation of VSMCs, autophagy can play an opposite role within diverse circumstances. Besides the above, two independent teams approved that IV application of astragaloside effectively inhibit autophagy and mineralization in VSMCs, in which the inhibition is accomplished within the involvements of the ERK signaling pathway mediated by lncRNA H19 and DUSP5 [53] and mainly associated with mesenchymal stromal cells (MSCs) [25]. The biological circumstances on osteogenic differentiation left a critical challenge for drug design when developing anti-VC targeting autophagy pathway.

## Autophagy affects vascular calcification by inhibiting apoptosis

It was proved that apoptotic cells can compose a nidus for the deposition of calcium-phosphate crystals [54]. The studies that apoptotic bodies form a nidus to nucleate apatite from dying VSMC [16] reflect the importance of VC promoted apoptosis, which indicated a possible mechanisms initiating the VSMC calcification process [55].

### Autophagy inhibits apoptosis and vascular calcification

Many works linked apoptosis and VSMC calcification in both human sample and animal models. For instance, massive apoptotic cell death found in both human and animal atherosclerotic plaques [56], suggests that apoptosis could promote calcification of providing matrix through the release of apoptotic bodies in nidus along with nucleation sites of VC. In calcification model of uremia, apoptosis is positively detected with calcification of VSMCs, which always occurs before calcification. The observation exhibited that in which apoptotic bodies were normally found to encompass with high concentrations of calcium by accumulating on extracellular matrix (ECM), and eventually leading to calcification [57].

In hyperphosphate-induced VC, inhibition of calcium aggradation can be attained by inhibiting apoptosis and potentiating autophagy and many hormonal molecules exhibits their critical roles [58–61]. Melatonin protected VSMCs against apoptosis and attenuated β-GP-induced VSMC calcification via autophagy stimulation associated to an AMPK/mTOR/ULK1 signaling pathway [59]. Bavachin suppresses apoptosis and calcification effects in HASMCs. Mechanism of the above hormonal effect is dependent on Atg7/mTOR-mediated autophagy pathway and suppression of Wnt/β-catenin signaling [60]. Cozzolino group also claimed the related role of autophagy and apoptosis in the iron citrate preventing calcium deposition in high Pi-calcified VSMC associated with an iron-induced positive modulation of GAS6/AXL synthesis. They demonstrated that iron citrate arrests further high Pi-induced calcium deposition through an anti-apoptotic action and induction of autophagy on established calcified VSMC [61].

While a different in vitro model was established to delay high phosphate-induced VC progression, the group treated rat aortic VSMCs with high Pi in a repeated and short suspensions of high Pi treatment (intermittent suspension, IS). The treatment generates significant inhibition on high Pi calcification. Their data further approved that the inhibition on apoptosis is carried out through the preservation of AXL protein levels [62] and enhanced autophagy plays a protective role in arterial calcification through inhibiting apoptosis.

### Mitophagy partially reverse mitochondrial disorder of vascular calcification

After mitochondrial toxicity is induced, the damaged mitochondria will be wrapped in a double-layer membrane structure to form autophagosomes, which are further degraded by lysosomes, otherwise the intrinsic apoptotic pathways will be activated. Its on-site accumulation promotes crystallization as nucleation of calcium phosphate crystals for further VC plaques [63]. Other data demonstrated that lactate impaired mitochondrial function inducing oxidative stress and apoptosis during VSMC calcification.

The Drp1‑related mitochondrial fission promoted by lactate through NR4A1 upregulation, while NR4A1 suppressed the autophagic flux and BNIP3‑mediated mitophagy, which were by p53 regulated phosphorylation. The regulation of Drp1 and BNIP3 is further related to the NR4A1/DNA‑PKcs/p53 pathway in the pathological plaques [64]. BNIP3-mediated mitophagy could partially reverse mitochondrial disorder, excessive oxidative stress and enhanced apoptosis, which plays a protective role against VSMC calcification in the presence of lactate [65]. This phenomenon suggests that, to some extent, autophagy/mitophagy can avert the activation of apoptotic pathways by the removal of damaged mitochondria [66].

As two critical catabolic processes that assist preserve cell and tissue homeostasis [67, 68], autophagy and apoptosis are highly related in deciding cell fate. Apoptosis stringently related to autophagy could be considered the result of the failure of autophagy to re-establish a physiological balance for the cells involving in survival. Autophagy can promote cell survival, but under certain conditions, autophagy also protected cells from necrosis via promoting apoptosis. The autophagy played a role in either promoting apoptosis or inhibiting apoptosis [68]. Therefore, the precarious issue is how to determine the range of moderate and appropriate mitophagy for VC.

## VC associated osteogenic resembling and cellular pathological autophagy

The normal distribution where calcium is mineralized in the human body are bones and teeth meanwhile it becomes VC when calcium is excessively deposited on the blood vessel wall [28]. In the process of VC, the cells within vascular wall are transformed into an osteoblast-like phenotype, and begin to synthesize and secrete a variety of proteins related to bone formation, such as ALP, bone morphogenetic protein (BMP), osteopontin (OPN), osteonectin, osteocalcin, etc. [69]. Calcium nodules are formed in the extracellular matrix or cytoplasm of these cells, which are tightly associated with non-bone osteogenesis or autophagy related mineral resembling [70].

### Vascular calcification and osteoporosis

While VC patients with high risk are often accompanied with osteoporosis, Matrix Gla protein (MGP) and osteocalcin are important factors for their regulation. The normal calcium balance in the human body guarantees the amount of calcium required in bones and teeth, but not abnormal calcium deposition in other locations such as blood vessels and internal organs [71]. When this calcium associated metabolism is disturbed, it will lead to a series of diseases, such as osteoporosis due to excessive loss of calcium in the bones, and dominant gain of calcium on the blood vessel wall.

VC often occurs simultaneously with low bone mineral density or poor bone turnover [72]. In general consideration, the balance and metabolism of calcium are closely related to vitamin K2, vitamin D3, MGP and osteocalcin [73]. The study of VC mechanism often involves changes in the expression of proteins related to bone development, such as OPG/RANK, OPN, MGP, BMP, etc. [69], in which the proteins plus blood calcium are cellular and mechanically resembled bone tissue architecture during osteogenesis. This bone-like structure formation is based on the structure and scaffold of blood vessels, and depending on osteoblasts differentiated from angiogenic pericytes or blood-borne mesenchymal cells surrounding the vasculature [74]. Up to date, many factors regulating bone mineralization are proved in calcified plaques [75, 76].

### Autophagy exhibits variable effects in the metabolism of bone tissue and vessel

Property of autophagic recycling damaged organelles is highly related to bone metabolism during the dynamic synthesis and degradation process in bone [77]. Autophagy can display variable effects in the calcification of chondrocytes/osteocytes. Then, AdipoRon can activate autophagy of Osteoarthritis (OA) chondrocytes through the AMPK mTOR pathway, and as well improve autophagy contribution to suppress calcification in OA chondrocytes [78].

Using osteocyte differentiation approach, Vrahnas and colleagues collected data within in vitro system on murine stromal cell lines, Ocy454 cells and Kusa 4b10 cells compared to Dmp1Cre mice (Tg(Dmp1Cre)^1Jqfe^) and EphrinB2-floxed (Efnb2^tm1And^) mice. The data exhibited that significant mineral deposition in Dmp1Cre.Efnb2^f/f^ bone along with massive increase of autophagosomes [79] and EphrinB2 deletion in osteocytes generated defected mice with brittle bones. Their result approved that Osteocytic EphrinB2 could limits autophagy through RhoA, which could be responsible for limiting mineral accumulation and carbonate substitution within the bioapatite matrix and restraining collagen fiber compaction [79]. Subsequently, further investigations unveiled that EphrinB2 deficiency in bones dysregulated many genes including *Fam134b* [80], *Fbxo32* [81], *Lama2* [82], *Bnip3* [83], *Peg3* [84], *Eps8l1* [85], *Klf1* [86], *Tspo2* [87], and *Unc5a* [88], which is specifically related to a series of autophagy processes, including mitophagy and ER-phagy.

Recently, a study claimed that autophagy plays a critical role in promoting vascular calcification within osteoblast differentiation and mineralization using vitamin K2-induced MC3T3 E1 cells [89]. During post-natal bone development, autophagy is induced in growth-plate chondrocytes during post-natal bone development via regulating the secretion of type II collagen (Col2). When FGFR4 and JNK-dependent activation, the autophagy initiated complex VPS34 and beclin-1 [90]. Therefore, on compression on how autophagy regulating VC, further investigations can plagiarize the above information from bone formation [80–88].

## The effect of autophagy regulation in stem cells on vascular calcification

Because both VSMCs and osteoblasts are mesenchymal originated, many works focus on the mechanism of VC on the correlation with stem cells. Interestingly, as MSCs have a dual role as progenitors to osteoblasts and pericytes further to develop to VSMC, some studies found that cells with MSC phenotype in the adventitia of arteries are the major source of osteoblast-like cells in intimal and medial calcification [29]. The uremic milieu causes osteoblastic differentiation of MSC and calcification [91], indicating loss of vascular progenitor properties.

Recently, Carracedo and his colleagues discovered that initiated autophagy in calcific aortic valve stenosis (CAVS) confers protection of valvular interstitial cells (VICs). Their data suggest that the upregulation of autophagy observed in the calcified tissue of these valves serves as a compensatory and pro-survival mechanism to protect valves from calcification [92]. Their results are also supported by another result that VIC autophagy could prevent calcification via pro-osteogenic signaling [93]. Hegner and colleagues discovered that mTORC1 and mTORC2 pathways show different regulatory roles in cell fate during the osteoblastic differentiation from MSCs. Furthermore, other studies demonstrated that blockade of autophagy can exacerbate calcification of differentiated MSC. Inhibition of AKT signaling or genetic depletion of mTORC2 abrogate the protective effect of rapamycin on MSC calcification. And enhanced mTORC2 signaling is sufficient in this protection effect from MSC against calcification [94]. Autophagy could be attributed a key role in the transition from undifferentiated MSC to osteoblast-like calcifying cells.

In a recent report, a study shows that, in tendon-derived stem cells (TDSCs), pioglitazone increased the ratio of LC3B/LC3A and decreased that of P62 expression, and performed as an agonist to promote autophagy via modulation of the AMPK/mTOR pathway. Pioglitazone treatment can induce autophagy flux in AGEs-treated TDSCs, which possesses anti-apoptosis, anti-senescence, and anti-ossification effects [95]. Based on the discussion above, targeting on differentiated progenitor cells such as VSMC and osteoblast-like cells could maintain and resort the endogenously physiological MSC function. Enabling protective cell fate patterns in the MSC-pericyte-VSMC-continuum could be an innovative approach for treatment of VC.

## MV involved in autophagy regulation of vascular calcification

As an effective approach based on recent investigation, MV can be transmitted among co-cultured cells through endocytosis and induced cell-cell communication. When extracellular MV exosomes containing low fetuin-A content were added to recipient VSMC, the calcification was increased upon these cells. The increase in calcium induced by cellular derived MV is partly attributable to the activation of NOX and MAPK (MEK1 and Erk1/2) signaling pathway [96].

MVs formed from VSMCs and macrophages under atherosclerotic conditions mainly, are calcified and released into the collagen-rich matrix within the intima of vessel [19, 97]. Macrophages indirectly promote mineral formation by producing the inflammatory cytokines while lipid oxidation products promote vascular cell mineralization. Xu, *et al* emphasized that autophagy would be an endogenous protective mechanism counteracting VC under hyperphosphatemia. The autophagy inhibition leads to increased MV release rather than cell apoptosis, and the inhibition promoted Pi-induced MV release and increased ALP activity may be the cause of calcification [31]. Other study on the calcification of aortic leaflets claimed that autophagy resulted in the release of MVs in early degenerative aortic valves, which attract inflammatory cells and triggered calcification of the valve [98, 99].

Depending on various induction signals, the origin and selected content in the MVs of autophagy promoted VSMC exocytosis are different [31, 32], and MVs are considered the first nidus for mineralization in the vessel wall [30]. Therefore, as an effective hydroxyapatite mediator, the mechanism of MV autophagy release in the initiation and development of VC needs more detailed research.

## Current clinical trial involved with autophagy related treatment for VC

Autophagy related treatment have been used for halting many cancer development involved with autophagic pathways including in both animal models and human samples [100–103]. Strong potential drugs against diverse tumors were designed and applied as anti-cancer therapy. Autophagy-targeting drugs such as autophagy inhibitors Choloroquine and Bafilomycin A1 targeting on endosomal acidification, and 3-Methyladenine and LY294002 targeting PI3K pathway currently approved for use in the treatment of solid and non-solid malignancies [100]. However, up to date, only one drug SNF472 against autophagy recently were used on Phase 2 treatment for treatment against cardiovascular calcification in patients with actual strong positive results [103, 104].

As a derivative of phytic acid, SNF472 (hexasodium salt of phytate) play a critical role as a potential treatment for Alzheimer's disease targeting on autophagy- associated proteins (beclin-1 and LC3B) [105]. Currently, using SNF472 as a calcification inhibitor, a clinical Phase II CaLIPSO trial (EudraCT 2016–002834–59) for the treatment of cardiovascular calcification is completed. Based on double-blind and placebo-controlled Phase II trial, computed tomography scan unveiled that, in randomized three groups including SNF472 300 mg (n=92)/SNF472 600 mg (n=91)/or placebo control (n=91), two SNF472 dosages significantly slowed down of both the accumulation of coronary artery calcium and the development of aortic valve calcification [103, 104]. This favorable data highlights strong possibility to develop more practical and high efficient approach with other drugs targeting on the pathway of the molecular mechanism of autophagy in VC associated cardiovascular disorders.

With the above promising results, it would be rational to accomplish more clinical trials associated with autophagic proteins (Table 2) based on massive positive data obtained animal models. Providentially, along with endosomal acidification targeted and PI3K pathway targeted autophagy inhibitors, MAPK pathway associated inhibitors including SB202190 and SB203580 also be available for diverse VC related trials. The advantage should be employed in which many autophagy inhibitors are FDA approved drugs that can be used in other diseases [100–102].

**Table 2:** Autophagy related proteins in human and their functions in vascular calcification

#	Autophagy proteins involved	Functions in VC process	PubMed ID	Chromosome location/band	Transcript (bp)	CDS (bp)	Peptides (AA)	References
1	LC3-I transcript variant 1	Cysteine protease cleaved LC3 I from LC3 inhibits osteogenic differentiation of VSMCs	NM_032514	Chromosome 20: 34,546,854–34,560,345 forward strand; 20q11.22	964	366	121	Peng et al. [28];
	LC3-I transcript variant 2		NM_181509		971	378	125	Zhang et al. [34]; Song et al. [47]; Xu et al. [90]
2	Atg5(Autophagy related 5)	Knocking down Atg5 expression significantly upregulates β-GP-induced Runx2 expression and ALP activity along inhibition of osteogenic differentiation of VSMCs	JQ924061.1	Chromosome 6: 106,045,423–106,325,791 reverse strand; 6q21	828	828	275	Dai et al. [25]; Zhang et al. [34]
3	LC3-II	Along with enhancing autophagic flux, LC3-II augmenter the number of autophagosome by reducing the RUNX2 expression	NM_022818	Chromosome 16: 87,383,953–87,404,779 forward strand; 16q24.2	2147	378	125	Peng et al. [28]; Yang et al. [30]; Song et al. [47]; Xu et al. [90]
4	p62 (SQSTM1)	By reducing the RUNX2 expression, p62 augmenter the number of autophagosome	M88108	Chromosome 5: 179,806,398–179,838,078 forward strand; 5q35.3	2685	1332	443	Yang et al. [30]
5	Beclin1 transcript variant 1	As an up-regulator of autophagic pathway, beclin1 decreases expression of Runx2 and Msx2	NM_003766	Chromosome 17: 42,810,134–42,833,350 reverse strand; 17q21.31	2131	1353	450	Xu et al. [31]; Wang et al. [43]
			NM_001313998		2109	1353	450	
			NM_001313999		1840	1068	355	
6	Atg7 transcript variant 4	As an upstream autophagy-related gene of LC3, Atg7 promotes the conversion of LC3-I to LC3-II and prevention of calcification	NM_001349232	Chromosome 3: 11,272,309–11,557,665 forward strand; 3p25.3	5333	2112	703	He et al. [58]
	Atg7 transcript variant 5		NM_001349233		5087	2112	703	
	Atg7 transcript variant 7		NM_001349235		5224	2112	703	
7	VPS34 transcript variant 1	Forming VPS34–beclin-1 complex for autophagy initiation and promoting vascular calcification	NM_002647	Chromosome 18: 41,955,234–42,087,830 forward strand; 18q12.3	9415	2664	887	Cinque et al. [85]
	VPS34 transcript variant 2		NM_001308020		9226	2475	824	

## Conclusion and perspective

The maintenance of the normal structure of blood vessels and the regulation of its functions are critical for circulation system which is tightly related to autophagy. Within a certain criteria, autophagy activation as a protective affection on VSMCs can promote cell survival, lead to enhanced cell proliferation, migration, and extracellular matrix secretion, and reduce calcification. Removing cell debris such as misfolded proteins and dysfunctional organelles that can cause senescence and apoptosis, autophagy plays an important role in maintaining the adaptability of juvenile cells [95]. Reversely, when autophagy is inhibited, lack of autophagy leads to accumulation of harmful substances in cells, cell aging, changes in vascular structure, vasomotor function becomes abnormal, and the increasing incidence of VC. When autophagy is beyond the scope of its beneficial effects and/or over-activated, cell and organelle damage within VSMCs could lead to the vascular calcification accompanying with triggering cell death, and further accelerating the occurrence of VC. Autophagy plays an intricate and often distinct role under various pathological conditions.

The effects of autophagy on VC appears to be complicated, which depend on degree of autophagy associated with disease status, location, and the surrounding microenvironment. Autophagy activation in the presence of acute pathological damage is generally considered to be protective, resulting in degradation of dysfunctional cellular components and maintenance of cell homeostasis. Reversely, some chronic diseases induce sustained autophagy which may be detrimental, since defective autophagy can activate the apoptotic pathway, damage important organelles and cause cell apoptosis. In order to develop effective and unique approaches to slow down or eliminate VC targeting on VC-related autophagy, the functional and regulatory genes in osteogenesis including the bone formation such as *Fam134b*, *Klf1* and *Bnip3, and* the MSCs differentiation such as ALP, Runx2 and BMPs could be efficient candidates. The more intensive investigation on precise chick-point between these beneficial function and apoptotic death occurred on vessel wall, would be critical in further study in this field.

## Data Availability

Data and materials are available upon request to corresponding author.

## Abbreviations

AKT/PKB: Protein kinase B
AMPK: AMP-activated protein kinase
Atg: Autophagy-related gene
ANCR: Anti-differentiation noncoding RNA
AGEs: Advanced glycation end products
ALP: Alkaline phosphatase
apoE: Apolipoprotein E
α-SMA: Alpha smooth muscle actin
BMP: Bone morphogenetic protein
BSA: Bovine serum albumin
BNIP3: BCL2/adenovirus E1B 19 kDa protein-interacting protein 3
β-GP: β-Glycerophosphate
CREB: CAMP response element-binding protein
Col2: Type II collagen
CAVS: Calcific aortic valve stenosis
CKD: Chronic kidney disease
DUSP5: Dual specificity protein phosphatase 5
Drp1: Dynamin-related protein 1
DNA‑PKcs: DNA-dependent protein kinase, catalytic subunit
ERα: Oestrogen receptor alpha
ERβ: Oestrogen receptor beta
ER-phagy: Microautophagic degradation of the ER
ECM: Extracellular matrix
ERK1/2: Extracellular signal-regulated kinases1/2
FGF23: Fibroblast Growth Factor 23
FGF18: Fibroblast growth factor 18
FGFR4: Fibroblast growth factor receptor 4
GFP: Green fluorescent protein
GAS6: Growth arrest-specific 6
GHSR (ghrelin receptor): Growth hormone secretagogue receptor
HIF-1α: Hypoxia-inducible factor 1-alpha
HASMCs: Human Aortic Smooth Muscle Cells
IS: Intermittent suspension
JNK-dependent: C-Jun N-terminal kinases
LC3: Microtubule-associated protein 1A/1B-light chain 3
LAMP-1: Lysosomal-associated membrane protein 1
lncRNA H19: Long non-coding RNAs
MAPK: Mitogen-activated protein kinase
mTOR: Mammalian target of rapamycin
mRFP: Monomeric red fluorescent proteins
Mitophagy: Degradation of mitochondria
MScs: Mesenchymal stromal cells
mTORC1: Mammalian target of rapamycin complex 1
mTORC2: Mammalian target of rapamycin complex 2
MV: Matrix Vesicle
MMP: Mitochondrial membrane potential
MGP: Matrix Gla protein
NR4A1: Nuclear receptor subfamily 4 group A member 1
NOX: NADPH oxidase
OPN: Osteopontin
OCN: Osteocalcin
OPG: Osteoprotegerin
OA: Osteoarthriti
PI3K: Phosphoinositide 3-kinases
p53: Tumor protein p53
PDK4: Pyruvate dehydrogenase kinase 4
PPi: Pyrophosphoric acid
p62/SQSTM1: Sequestosome 1
ULK: Unc-51-like kinase 1
p53: Tumor protein p53
RANK: Receptor activator of nuclear factor κB
RhoA: Ras homolog family member A
Runx2: Runt-related transcription factor 2
SM22α: Smooth muscle 22 alpha
TGF-β1: Transforming growth factor beta 1
TEM: Transmission electron microscopy
TDSCs: Tendon-derived stem cells
VC: Vascular calcification
VSMCs: Vascular smooth muscle cells
VICs: Valvular interstitial cells
3-MA: 3-Methyladenine
